# MicroRNA-106a-5p Inhibited C2C12 Myogenesis via Targeting PIK3R1 and Modulating the PI3K/AKT Signaling

**DOI:** 10.3390/genes9070333

**Published:** 2018-07-02

**Authors:** Xiao Li, Youbo Zhu, Huifang Zhang, Guangjun Ma, Guofang Wu, Aoqi Xiang, Xin’E. Shi, Gong She Yang, Shiduo Sun

**Affiliations:** 1 Laboratory of Animal Fat Deposition and Muscle Development, College of Animal Science and Technology, Northwest A and F University, Yangling 712100, China; nicelixiao@nwsuaf.edu.cn (X.L.); zhuyoubo_2015@nwsuaf.edu.cn (Y.Z.); 13135587517@163.com (H.Z.); Guangjunma0904@126.com (G.M.); aoqixiang0305@126.com (A.X.); xineshi@nwsuaf.edu.cn (X.E.S.); gsyang999@hotmail.com (G.S.Y.); 2 Stake Key Laboratory of Plateau Ecology and Agriculture, Qinghai Academy of Animal Science and Veterinary Medicine, Qinghai University, Qinghai 810000, China; letitbe521@163.com

**Keywords:** miR-106a-5p, myogenic differentiation, muscle atrophy, PIK3R1, C2C12 cell line

## Abstract

The microRNA (miR)-17 family is widely expressed in mammalian tissues and play important roles in various physiological and pathological processes. Here, the functions of miR-106a-5p, a member of miR-17 family, were explored during myogenic differentiation in C2C12 cell line. First, miR-106a-5p was found to be relatively lower expressed in two-month skeletal muscle tissues and gradually decreased upon myogenic stimuli. Forced expression of miR-106a-5p significantly reduced the differentiation index, fusion index as well as the expression of myogenic markers (MyoD, MyoG, MyHC, Myomixer, Myomarker). Meanwhile, the levels of phosphorylated AKT were reduced by overexpression of miR-106a-5p, and administration of insulin-like growth factor 1 (IGF1), a booster of myogenic differentiation, could recover all the inhibitory effects above of miR-106a-5p. Furthermore, miR-106a-5p was elevated in aged muscles and dexamethasone (DEX)-treated myotubes, and up-regulation of miR-106a-5p significantly reduced the diameters of myotubes accompanied with increased levels of muscular atrophy genes and decreased PI3K/AKT activities. Finally, miR-106a-5p was demonstrated to directly bind to the 3’-UTR of PIK3R1, thus, repress the PI3K/AKT signaling.

## 1. Introduction

Skeletal muscle comprises approximately 50% of the body’s weight and is an endocrine and paracrine organ that plays a key role in the maintenance of the internal environment and homeostasis [1]. Myogenesis is an ordered and extremely complicated process, including myoblasts proliferation, withdrawal from the cell cycle, fusion into multinucleated myotubes, and hypotrophy/atrophy [2]. During muscle development, Myf5, MyoD and MRF4 are myogenic determination factors, and Myogenin is a downstream effector of MyoD and MRF4, and activate the myogenic differentiation program [3,4]. It has been reported that many pathways are involved in myogenesis, such as Wnt/β-Catenin Signaling [5], the TGFβ signaling pathway [6,7], JAK/STAT signaling pathway [8], and PI3K/AKT signaling pathway [9,10]. Recently, more and more microRNAs (miRs) have been reported to participate in myogenesis, such as miR-127 [11], miR-133 [12], miR-1/206 [13], and miR-432 [14]. However, there are still many miRs that remain to be discovered in myoblasts differentiation and muscle formation.

MiR is a class of evolutionarily conserved, regulatory noncoding RNAs of 18–24 nucleotides that participate in the fine-tuning of many, if not all, fundamental biological processes, including skeletal muscle development and muscle-related diseases [15,16]. MiRs have been exquisitely described to control gene expression by binding to mRNA targets using the seed sequences, and miRs with the same or similar seed sequences and origin are grouped into one family or cluster [15]. The miR-17 family consists of the miR-17-92 cluster, miR-106a-363 cluster and miR-106b-25 cluster [17], and the miR-17 family has been well documented to influence the survival, differentiation and functions of various kinds of mammalian cells [18,19]. However, the effects of miRs associated with the miR-17 family in myogenesis are controversial. MiR-17, miR-20a and miR-92a are reported to strongly repress myoblasts differentiation by targeting Enigma homolog 1 (*ENH1)*/Inhibitor of differentiation 1 (*Id1*) [20]. Over the same period, Luo reported that miR-20a/b promoted quail muscle clone 7 (QM-7) myoblasts differentiation by negatively regulating *E2F1* [21]. Intriguingly, miR-106a-5p, a member of the miR-106a-363 cluster, was reported to down-regulate during myogenic differentiation [22,23], and its role in myogenic differentiation deserves to be analyzed.

PI3K (p85α), encoded by *PIK3R1* gene, is a key protein involved in the PI3K/AKT signaling pathway [24], which is essential for myogenic differentiation [25,26]. It has been recently shown that PI3K/AKT signaling controls muscle-abundant miRs (myomiR) maturation during C2C12 myoblasts differentiation [27]. Insulin and insulin-like growth factor 1 (IGF1) are known as physiological activators of PI3K/AKT signaling in different cell types, including C2C12 myoblasts [28]. Administration of IGF1 promotes myoblast proliferation, differentiation [25,29], and induces myotube hypertrophy [30] by activating the PI3K/AKT signaling pathway. However, regulation of *PIK3R1* by miR-106a-5p and how miR-106a-5p responds to IGF1 stimuli to regulate myogenesis are still poorly understood.

In this study, we analyzed the expression profiles of miR-106a-5p and determined its role and mechanism on myogenesis. Our study identified miR-106a-5p as a novel negative regulator for myogenesis, and miR-106a-5p could repress differentiation and promote atrophy by blocking the PI3K/AKT signaling pathway through targeting *PIK3R1*.

## 2. Materials and Methods

### 2.1. Ethics Statement

All experimental mice were operated on in accordance with approved guidelines of the Animal Care and Use Committee of the Northwest A and F University, Yangling, China (NWAFU-314020038) and the guidelines of the Animal Use Committee of the Chinese Ministry of Agriculture (Beijing, China). Furthermore, C2C12 and HEK29T cell lines were obtained from American Type Culture Collection (ATCC, Manassas, VA, USA).

### 2.2. Cell Culture

The C2C12 myoblasts (ATCC, USA) were used to determine the function of miR-106a-5p during myogenesis. HEK293T (ATCC, USA) was employed for the luciferase reporter analysis. Cells were cultured in Dulbecco’s Modified Eagle Media (DMEM, Gibco, ThermoFisher, Waltham, MA, USA) supplemented with 10% FBS (Gibco) and 100 IU/mL penicillin-streptomycin at 37 °C with humidified 5% CO_2_ atmosphere. Upon shifting to a 2% horse serum containing medium, C2C12 myoblasts were induced to fusion and differentiation. The medium was changed every day.

### 2.3. Animals

The C57BL/6 male mice were purchased from the Fourth Military Medical University Animal Center (Xi’an, China) and raised in a controlled temperature (25 ± 1 °C) with a 12 h light/12 h dark cycle. Tissues were collected from two-month and six-month old mice. All procedures with mice were in accordance with approved guidelines of the Animal Care and Use Committee of the Northwest A and F University.

### 2.4. Transfections and Treatment of Myoblasts and Myotubes

To test the effects of miR-106a-5p on differentiating cells, myoblasts were seeded in 6-well or 12-well plates and transfected with 50 nM FAM-labeled miR-106a-5p agomir or negative control (GenePharm, Shanghai, China) using Lipo Plus (Sagecreation, Beijing, China) in Opti-EME (Gibco) according to the manufacturer’s instructions. Furthermore, 75 nM IGF1 recombinant protein (Sino Biological, Beijing, China), a PI3K-AKT signaling activator, was used to recover the effects of miR-106a-5p.

To test the effects of miR-106a-5p on well-differentiated myotubes (5 days post-differentiation), myotubes were incubated with 50 μM Dexamethasone (DEX) for 36 h, then cells were harvested for further analysis. Furthermore, myotube transfection (50 nM miR-106a-5p agomir or negative control) was performed with Lipo Plus (Sagecreation) according to the manufacturer’s instructions, and cells were harvested 36 h after transfection.

### 2.5. Real-Time Quantitative PCR

The total RNA was extracted with Trizol reagent (TakaRa, Ostu, Japan) as recommended by the manufacturer, and the concentration and quality were analyzed by the NanoDrop 2000 (ThermoFisher). Then complementary DNA (cDNA) was synthesized by reverse transcription kit (TakaRa). Real-Time quantitative PCR (RT-qPCR) was performed using Applied Biosystems qPCR instrument (ThermoFisher) and SYBR green PCR Master Mix (Vazyme, Nanjing, China). The expressions of all coding genes were normalized to β-actin, and U6 small RNA was the internal reference when testing the level of miR-106a-5p. miRs quantification was determined by using Bulge-loop^TM^ miRNA qRT-PCR Primer Set (one RT primer and a pair of qPCR primers in each set) specific for miR-106a-5p, designed and synthesized by RiboBio (Guangzhou, China) and other primers which were synthesized by Invitrogen (Shanghai, China). The sequences were shown in Table 1.

### 2.6. Western Blotting Analysis

Cells were harvested in radioimmunoprecipitation assay (RIPA) lysis buffer (Applygen Technologies Inc., Beijing, China) supplemented with protease and phosphatase inhibitor cocktail (Cwbiotech, Jiangsu, China). Protein concentration was determined by BCA Protein Assay Kit (Cwbiotech) and 25 μg proteins per sample were loaded and separated using a 5% stacking gel and a 10% separating gel. Separated proteins were transferred to PVDF membrane (CST, Boston, MA, USA), and then the membrane was blocked in 5% BSA buffer for 2 h at room temperature, and incubated with primary antibodies against MyoD (1:500, #NB100-56511SS, Novus Biologicals, Littleton, CO, USA), MyoG (1:500, #NB100-56510SS, Novus Biologicals), MyHC (1:1000, #MAB4470, R and D Systems, Minneapolis, MN, USA), p-PI3K (1:500, #4228S, CST, Danvers, MA, USA) and PI3K (p85α) (1:500, #4257S, CST), p-AKT (1:1000, #4257S, CST) and AKT (1:1000, #9272S, CST), p-mTOR (1:1000, #5536S, CST) and mTOR (1:1000, #2983S, CST), MAFbx (1:500, #sc-166806, Santa Cruz, Dallas, TX, USA), and MuRF1 (1:500, #C-11, Santa Cruz) at 4 °C overnight. After washing three times (10 min once) in TBST, membranes were incubated with HRP-conjugated goat anti-mouse IgG (1:3000, #BA1050, BosterBio, Wuhan, China) or goat anti-Rabbit IgG (1:3000, #BA1054, BosterBio) for 1.5 h at 4 °C. Imaging and quantification of the bands were carried out by Gel Doc XR system (Bio-Rad, Hercules, CA, USA) and Image Lab software (Bio-Rad).

### 2.7. Immunofluorescence Analysis

Differentiated C2C12 myotubes were fixed in 4% paraformaldehyde, permeabilized with 0.5% Triton X-100, and then blocked in 5% BSA for 30 min. Later, myotubes were sequentially incubated with anti-myosin heavy chain monoclonal antibody (1:200, #MAB4470, R and D Systems) overnight and an Alexa Fluor 594-conjugated anti-mouse IgG (1:1000, #SA00006-3, Proteintech, Chicago, IL, USA) for 1 h at 4 °C. Finally, nuclei were stained with DAPI. Images were captured with a fluorescence microscope (Nikon, Tokyo, Japan). The myotubes with 1–3, and > 4 nuclei were counted, respectively. The differentiation index was determined as the percentage of MyHC-positive nuclei among total nuclei, and the myotube fusion index was determined as the distribution of the nucleus number in total myotubes according to a previous report [31].

### 2.8. Luciferase Reporter Assays

The 3′-UTR of PIK3R1 including miR-106a-5p complementary sequences were synthesized by GENERABIOL (Chuzhou, Anhui, China), and inserted into psiCHECK^TM^-2 Vector (Promega, Madison, WI, USA). Wild-type or mutated constructs and miR-106a-5p agomir or negative control (NC) were co-transfected. Transfected cells were analyzed 48 h post transfection with Dual Luciferase Reporter Assay System (Promega) based on the instructions.

### 2.9. Statistical Analyses

Values are presented as the mean ± standard error of the mean (SEM), and statistical significance of differences was determined by Student’s *t*-test or one-way analysis of variance (ANOVA) by IBM SPSS Statistics 22.0 (Armonk, NY, USA). A *p*-value of < 0.05 was considered statistically significant.

## 3. Results

### 3.1. The Expression Profiles of miR-106a-5p in Mice

**Figure 1:**
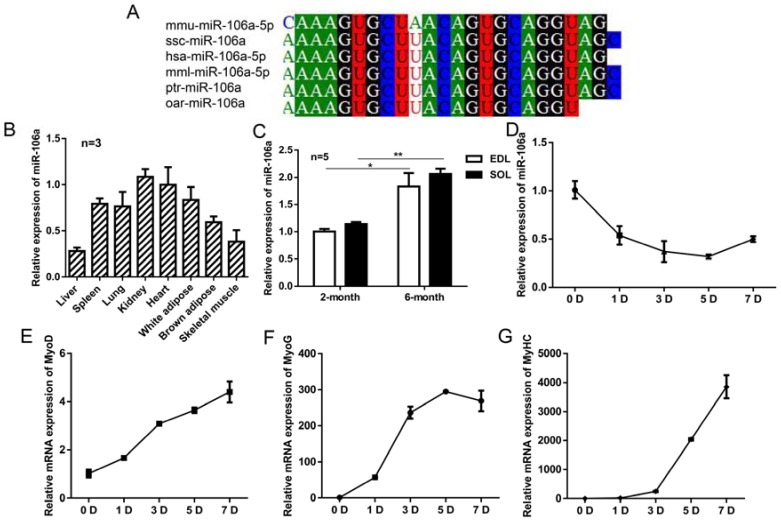
The profiles of miR-106a-5p in mice. (**A**) The homology comparison of microRNA (miR)-106a-5p between different species. mmu: *mus musculus*; ssc: *sus scrofa*; has: *homo sapiens*; mml: *macaca mulatta*; ptr: *pan troglodytes*; oar: *ovis aries*. Nucleotides with the same shadow were conserved across species. (**B**) The expression level of miR-106a-5p in different tissues of two-month-old mice. White adipose: subcutaneous white adipose; Skeletal muscle: tibialis anterior muscle. (**C**) The expression profiles of miR-106a-5p in skeletal muscles of two-month-old and six-month-old mice. EDL: extensor digitorum longus; SOL: soleus. (**D**–**G**) Real-time quantitative PCR (RT-qPCR) analysis of miR-106a-5p, MyoD, MyoG, MyHC expression during myoblast differentiation (*n* = 3 per group). D: days. Data were presented as mean ± standard error of the mean (SEM). * *p* < 0.05, ** *p* < 0.01.

MiR-106a-5p was highly conserved among detected species based on the nucleotide sequences (Figure 1A). MiR-106a-5p was widely expressed in various tissues in two-month mice, especially in heart and kidney, but a small amount of expression was detected in skeletal muscle (Figure 1B). Further, a significantly higher expression was found in Extensor Digitorum Longus (EDL) and Soleus muscle (SOL) of six-month old mice than that of two-month (Figure 1C). Furthermore, the expression of miR-106a-5p gradually decreased during myogenic differentiation in C2C12 cell line (Figure 1D), while the expression levels of myogenic markers MyoD, MyoG, and MyHC were significantly up-regulated (Figure 1E–G).

### 3.2. MiR-106a-5p Suppresses Myoblast Differentiation by Inhibiting the PI3K-AKT Signaling Pathway

**Figure 2:**
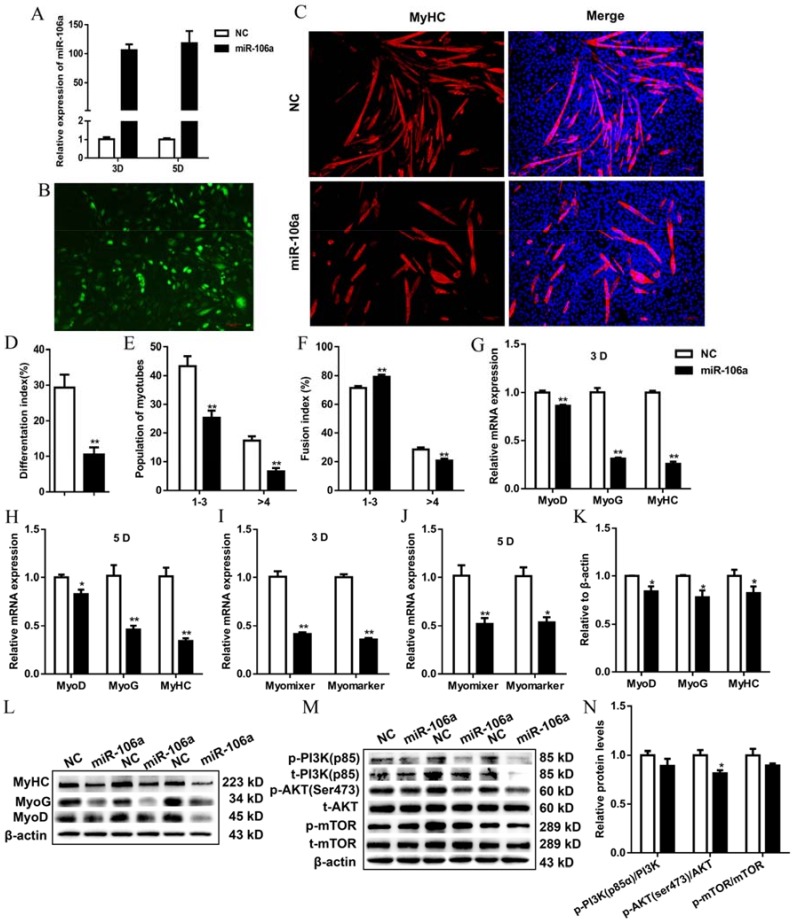
MiR-106a-5p inhibited the myogenic differentiation of C2C12 myoblasts. (**A**) Overexpression efficiency of miR-106a-5p 3 days (d) and 5 d post differentiation. NC: negative control; (**B**) The fluorescent microscopy images of C2C12 cells transfected with FAM-labeled miR-106a-5p agomir (×10). Scale bars = 500 μm; (**C**) Immunostaining for MyHC (red) and DAPI (blue) on 5 d post differentiation (×20). Scale bars = 100 μM; (**D**–**F**) The statistical results of differentiation index, fusion index and the populations of myotubes, respectively;1-3 indicates myotubes with 1, 2 or 3 nucleus, >4 indicates myotubes with 4 more nucleus; (**G**,**H**) The mRNA expression of MyoD, MyoG, MyHC on 3 d and 5 d post differentiation; (**I**,**J**) The mRNA expression of Myomarker and Myomixer 3 d and 5 d post differentiation; (**K**) The statistical results of MyoD, MyoG, MyHC proteins in Figure 2L; (**L**) Western blot analyzed for MyoD, MyoG, MyHC proteins 5 d post differentiation; (**M**) Protein levels of key molecules in PI3K-AKT pathway in C2C12 cells transfected with miR-106a-5p agomir or NC on 5 d post differentiation; (**N**) The statistical analysis of phosphorylated PI3K (p85α), AKT (sre473) and mTOR (ser2448). Data were presented as mean ± SEM. *n* = 3 per group. * *p* < 0.05, ** *p* < 0.01.

Cells were transfected with FAM-labeled miR-106a-5p agomir when reaching 80~90% confluence and then induced to myogenic differentiation at full confluence. Transfection increased miR-106a-5p expression level by around 100-fold (Figure 2A), and almost all cells were FAM positive (Figure 2B). Overexpression of miR-106a-5p significantly decreased the quantity of MyHC positive cells (Figure 2C). In addition, the percentage of multinucleated myotubes, myotube size and myotube fusion index were significantly decreased in cells transfected with miR-106a-5p (Figure 2D–F). Moreover, myogenic regulatory factors (MyoD, MyoG, and MyHC) were down-regulated by miR-106a-5p agomir (Figure 2G–L). Consistently, the expression of fusion genes, Myomixer and Myomarker, were inhibited by enforced miR-106a-5p (Figure 2I,J). Furthermore, the phosphorylations of AKT (ser473) was significantly inhibited by miR-106a-5p agomir, although the phosphorylations of mTOR (ser2448) and PI3K (p85α) were not significantly changed (Figure 2M,N). Collectively, these results suggested that miR-106a-5p could interfere with C2C12 myoblast differentiation and fusion by blocking PI3K-AKT signaling.

**Figure 3:**
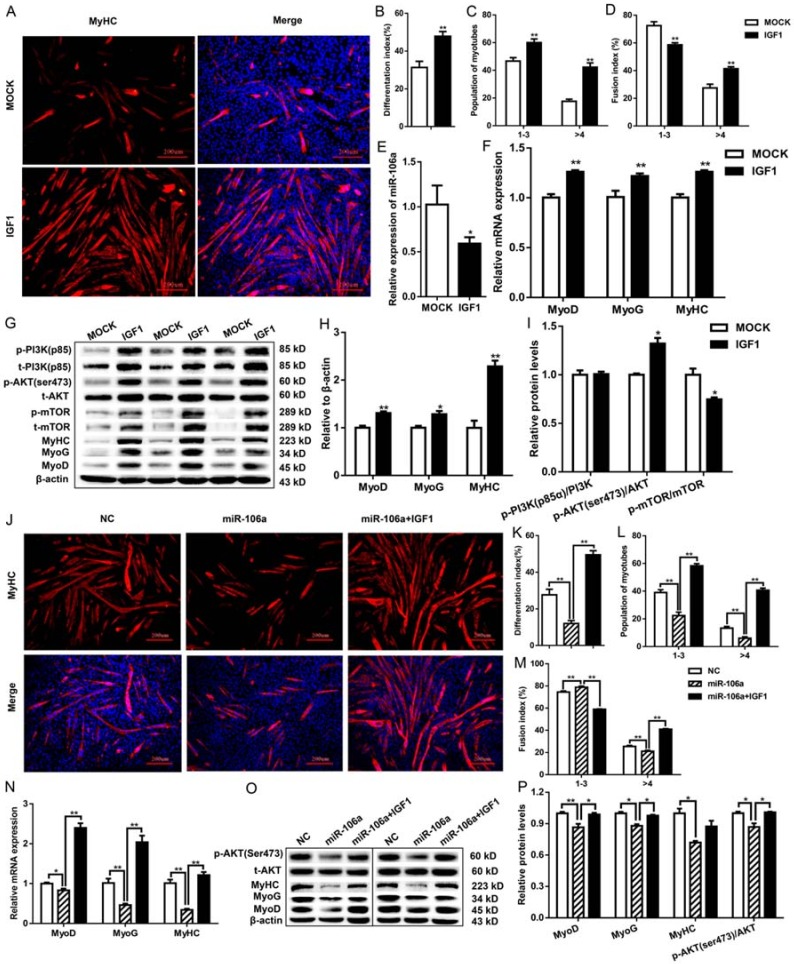
Insuline-like growth factor (IGF1) antagonized the effects of miR-106a-5p on myogenic differentiation in C2C12. All data were collected from C2C12 myotubes 5 d post differentiation. (**A**) MyHC immuno-staining of cells incubated in differentiation medium with or without 75 μM IGF1 recombinant protein. Scale bars = 200 μm; (**B**–**D**) The statistical results of differentiation index, fusion index and the populations of myotubes, respectively; (**E**) The expression of miR-106a-5p upon IGF1 stimuli; (**F**) The mRNA levels of MyoD, MyoG, MyHC in myoblasts cultured in differentiation medium containing 75 μM IGF1 recombinant protein or not; (**G**) Western-blot analysis of myogenic regulatory factors (MyoD, MyoG, MyHC) and key molecules in PI3K-AKT pathway in cells; (**H**,**I**) The statistical results of Figure 3G; (**J**) Immunostaining of MyHC (red) and DAPI (blue). Scale bars = 200 μm; (**K**–**M**) The statistical results of differentiation index, fusion index and the populations of myotubes, respectively; (**N**) The mRNA levels of MyoD, MyoG, MyHC; (**O**) Western blot analysis for MyoD, MyoG, MyHC and p-AKT/AKT upon IGF1 challenge; (**P**) Statistical analysis of MyoD, MyoG, MyHC and p-AKT/AKT levels in Figure 3O. Data were presented as mean ± SEM. *n* = 3 per group. * *p* < 0.05, ** *p* < 0.01.

In addition, treatment of 75 nM IGF1 recombinant proteins during myogenic differentiation significantly increased the number of myotubes, differentiation index, and multinucleated myotube fusion index (Figure 3A–D), and dramatically reduced the expression of miR-106a-5p (Figure 3E). Furthermore, IGF1 up-regulated the expression of MyoD, MyoG, and MyHC (Figure 3F–H), triggered the activation of PI3K/AKT signaling pathway, stimulated the phosphorylation of AKT (ser473) (Figure 3G,I). Notably, IGF1 fully restored miR-106a-5p-induced inhibitory effects on myogenic differentiation, suggested by the increased MyHC positive cells, differentiation index, and multinucleated myotube fusion index (Figure 3J,M) and up-regulated expression of MyoD, MyoG, MyHC (Figure 3N–P). Furthermore, the reduced AKT (ser473) phosphorylation induced by miR-106a-5p was also relieved by recombinant IGF1 protein (Figure 3O,P).

### 3.3. MiR-106a-5p Contributes to C2C12 Myotubes Atrophy by Suppressing PI3K-AKT Signaling Pathway

**Figure 4:**
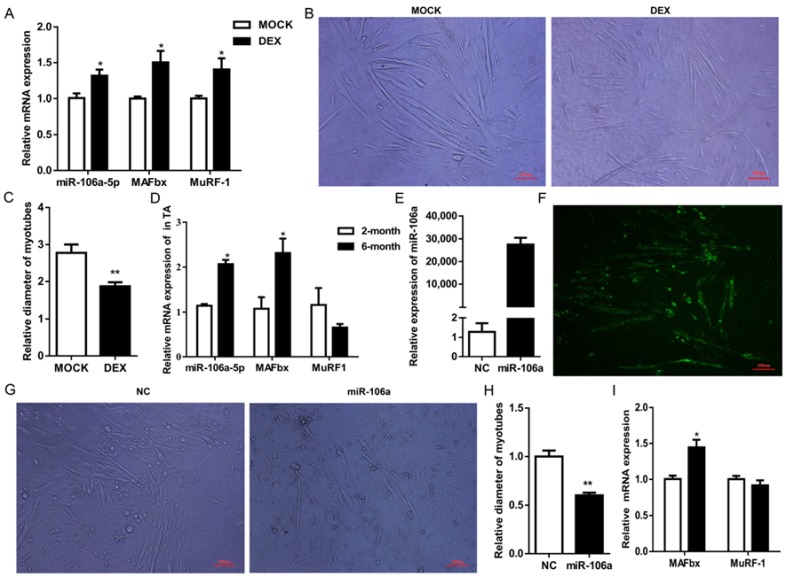

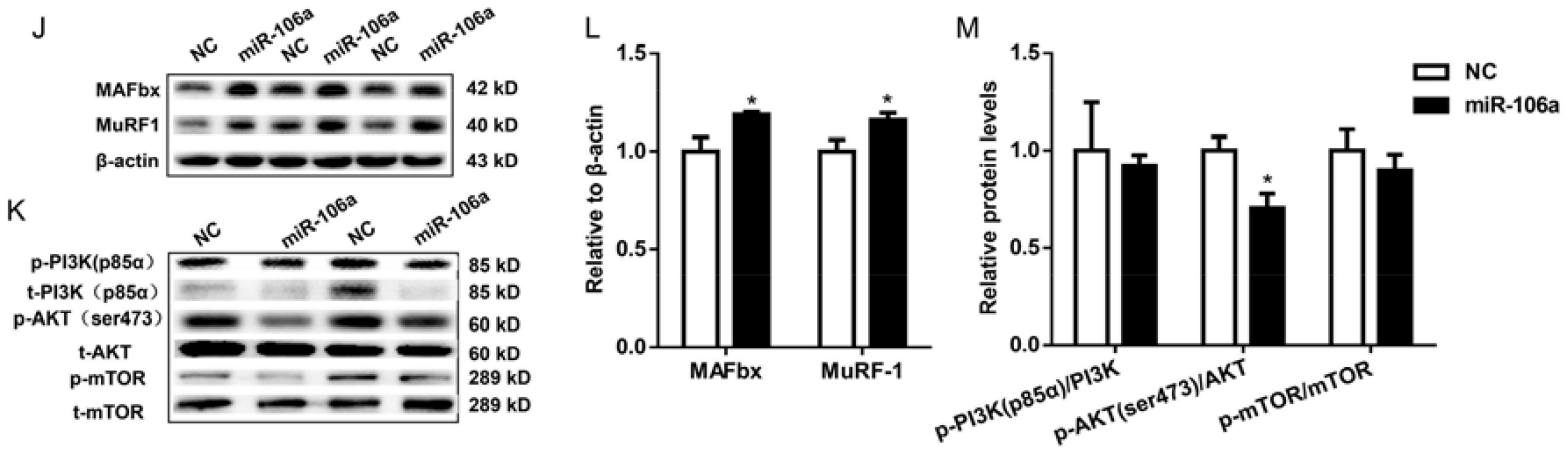
MiR-106a-5p contributed to myotubes atrophy. (**A**) Expression level of miR-106a-5p, MAFbx and MuRF1 mRNA after incubated with Dexamethasone (DEX) 36 h; (**B**) Morphological change of myotubes after DEX treatment 36 h (×20). Scale bars = 100 μm; (**C**) Relative diameter of myotube in Figure 4B; (**D**) RT-qPCR determined the expression of miR-106a-5p, MAFbx and MuRF1 in tibialis anterior (TA) muscle from two-month and six-month old mice (*n* = 5 per group); (**E**) Expression level of miR-106a-5p in C2C12 myotubes transfected with miR-106a-5p agomir or negative control for 36 h; (**F**) The fluorescent microscopy image of C2C12 cells transfected with FAM-labeled miR-106a-5p agomir. Scale bars= 100 μm; (**G**) The myotubes white light images after being transfected 36 h (×20); (**H**) Relative diameter of myotube from Figure 4G; (**I**) RT-qPCR analysis showed up-regulated MAFbx expression in C2C12 myotubes transfected with miR-106a-5p agomir 36 h; (**J**,**L**) Western blot showed that miR-106a-5p positively regulated analysis MAFbx and MuRF1 in C2C12 myotubes; (**K**,**M**) miR-106a-5p suppressed PI3K/AKT pathway in C2C12 differentiated myotubes. Results are presented as the mean ± SEM. *n* = 3 per group. * *p* < 0.05, ** *p* < 0.01.

Expressions of miR-106a-5p, as well as MAFbx and MuRF1 in C2C12 myotubes, were significantly elevated with the treatment of 50 μM DEX (Figure 4A). DEX significantly reduced the diameter of C2C12 myotubes (Figure 4B,C), indicating muscle atrophy. In addition, expressions of miR-106a-5p together with MAFbx, not MuRF1 were much higher in tibialis anterior (TA) muscles of six-month mice than that of two-month mice (Figure 4D).

In well-differentiated C2C12 myotubes, miR-106a-5p agomir was transfected to confirm its function in regulating myotubes atrophy. The overexpression efficiency of miR-106a-5p agomir was 30,000-fold higher than NC (Figure 4E), and the FAM-labeled miR-106a-5p agomir could be observed in almost all myotubes (Figure 4F). Moreover, enforced miR-106a-5p expression significantly decreased the diameter of C2C12 myotubes (Figure 4G,H) and increased the expression of MAFbx both at mRNA and protein levels (Figure 4I,J,L). The protein levels of another atrophy marker, MuRF-1 were also significantly enhanced by miR-106a-5p agomir (Figure 4J,L). Again, enhanced miR-106a-5p expression significantly repressed the phosphorylation of AKT (ser473) (Figure 4M). Together, these results indicated that miR-106a-5p promote C2C12 myotubes atrophy by repressing PI3K/AKT signaling pathway.

### 3.4. PI3K (p85α) Is a Target Gene of miR-106a-5p in Differentiating and Well-Differentiated C2C12 Cells

**Figure 5:**
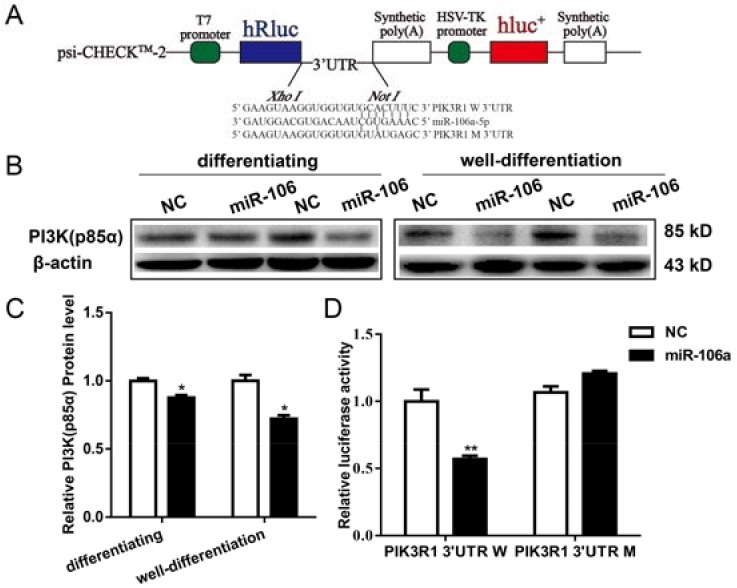
PI3KR1 was demonstrated to be a target of miR-106a-5p in C2C12 cells. (**A**) The schematic map of psiCHECK-PIK3R1 3′ UTR luciferase reporter constructs (hRluc: synthetic *Renilla* luciferase gene; HSV-TK: herpes simplex virus type 1 thymidine kinase promoter; hluc+: synthetic firefly luciferase gene; W: wild-type; M: mutant type); (**B**,**C**) Western blot analysis the expression of PI3K (p85α) protein (*n* = 3 per group); differentiating, 3 d post differentiation; well-differentiated, 36 h post transfection of myotubes; (**D**) The psiCHECK-PIK3R1 3′ UTR plasmid were co-transfected into 293 T-cells with miR-106a-5p agomir or NC. Luciferase activities were measured 48 h after transfection (*n* = 5 per group). Data were presented as mean ± SEM. * *p* < 0.05, ** *p* < 0.01.

*PIK3R1*, encoding PI3K (p85α) protein, is predicted to be a putative target of miR-106a-5p by TargetScan (http://www.targetscan.org/). Therefore, the 3′ untranslated region (3′UTR) of *PIK3R1* (W: wild-type; M: mutant type) was cloned into psi-CHECK^TM^*-2* backbone (Figure 5A). In addition, transfection of miR-106a-5p mimics significantly repressed the expression levels of PI3K (p85α) protein (Figure 5B,C) both in differentiating and well-differentiated C2C12 cells. Furthermore, luciferase assays confirmed that miR-106a-5p could bind to the wild-type of 3′UTR of *PIK3R1*, instead of the mutated one (Figure 5D).

## 4. Discussion

Previous studies reveal a critical requirement for miR-106a at the early mammalian development. MiR-106a is differentially expressed in developing mice embryos and functions to control differentiation of stem cells [32]. In addition, miR-106a is down-regulated in myoblasts differentiation [22,23]. In our study, miR-106a-5p was observed to decrease in a time-dependent manner during myogenic differentiation in C2C12 cells and is relatively lower expressed in adult skeletal muscle. These data indicate that miR-106a-5p might be a negative regulator for myogenesis. However, there was no significant difference in the expression of miR-106a-5p in fast EDL and slow SOL muscle, suggesting miR-106a-5p might have limited effects on muscle fiber type transition.

Given that miR-106a-5p was down-regulated in C2C12 myogenic differentiation, miR-106a-5p agomir was used to explore the effects of miR-106a-5p on myogenesis. Here, transfection of miR-106a-5p agomir significantly reduced the differentiation index, fusion index as well as the expression levels of myogenic markers, suggesting miR-106a-5p could dramatically repress myogenic differentiation. Meanwhile, enforced expression of miR-106a-5p significantly reduced the level of p-AKT (ser473) in C2C12 myoblasts. It has been well documented that PI3K/AKT is a crucial signaling pathway to promote myogenesis and induce muscle hypertrophy [33,34]. IGF1 is a stimulator of PI3K/AKT signaling during muscle differentiation [35]. In our study, administration of 75 nM IGF1 recombinant protein totally reversed the inhibitory effects of miR-106a-5p. Taken together, miR-106a-5p might interrupt C2C12 myoblasts differentiation by disrupting AKT activity.

Furthermore, miR-106a-5p is previously reported to be upregulated in limb-girdle muscular dystrophies types 2A and 2B and is involved in muscular disorders [36]. In the present study, miR-106a-5p accompanied with atrophic factors were up-regulated in aged muscles and DEX-treated myotubes, and overexpression of miR-106a-5p was sufficient to reduce the diameters of well-differentiated myotubes in vitro, indicating miR-106a-5p might be involved in muscle atrophy. During miR-106a-5p-induced muscle atrophy, decreased phosphorylated AKT (ser473) was observed. Similarly, miR-18a, a member of miR-17-92 cluster, is demonstrated to decrease during myogenic differentiation and promotes muscle atrophy by targeting IGF1 [37]. Collectively, our data indicate that miR-106a-5p might induce myotube atrophy through blocking the AKT signaling pathway.

In our study, pan-PI3K (p85α) protein levels were repressed by enforced miR-106a-5p agomir in both differentiating and well-differentiated myotubes. PI3K (p85α), encoded by *PIK3R1* gene, is a regulatory subunit of PI3Ks and essential for myoblasts proliferation and differentiation [24,38]. Germline deletion of the *PIK3R1* gene leads to impaired muscle growth, and a significant reduction in muscle weight and fiber size [39]. Moreover, *PI3KR1* was identified as the target of miR-128a [40] and miR-29b in muscle cells [41]. Here, the luciferase reporter assay showed that miR-106a-5p directly bound to the *PIK3R1* 3′ UTR, and *PI3KR1* is a novel target of miR-106a-5p in C2C12 cells.

In conclusion, miR-106a-5p is identified as a novel repressor of myogenesis, and miR-106a-5p represses differentiation and promotes atrophy by blocking the PI3K-AKT signaling pathway through targeting *PIK3R1*.

## Figures and Tables

**Table 1:** The primer sequences used for real-time quantitative PCR.

Name	Forward	Reverse
MyoD	CCACTCCGGGACATAGACTTG	AAAAGCGCAGGTCTGGTGAG
MyoG	GAGACATCCCCCTATTTCTACCA	GCTCAGTCCGCTCATAGCC
MyHC	GCGAATCGAGGCTCAGAACAA	GTAGTTCCGCCTTCGGTCTTG
Myomarker	CCTGCTGTCTCTCCCAAG	AGAACCAGTGGGTCCCTAA
Myomixer	GTTAGAACTGGTGAGCAGGAG	CCATCGGGAGCAATGGAA
MAFbx	CAGCTTCGTGAGCGACCTC	GGCAGTCGAGAAGTCCAGTC
MuRF1	GTGTGAGGTGCCTACTTGCTC	GCTCAGTCTTCTGTCCTTGGA
β-actin	GCCATGTACGTAGCCATCCA	ACGCTCGGTCAGGATCTTCA
